# About the right to be ill

**DOI:** 10.1007/s11019-017-9790-1

**Published:** 2017-07-14

**Authors:** Jacek Halasz

**Affiliations:** Department of History and Philosophy of Medical Science, Medical University of Gdansk, ul. M. Skłodowskiej-Curie 3a, 80-210 Gdańsk, Poland

**Keywords:** Medical ethics, Human rights, Disability, Healthism, Illness, History of medicine

## Abstract

The article raises the issue of ‘the right to be ill’, formulated by Tadeusz Kielanowski, a Polish physician and humanist. According to him, the right to health should be supplemented by the principle which would serve the protection of people with diseases or disabilities. One-sided interpretation of ‘the right to health’ may result in various forms of intolerance and discrimination. This paper presents what dangers Kielanowski recognized and explains why his approach was considered to be a novelty; what the idea of ‘the right to be ill’ is, how the need for it is substantiated. This idea is considered in the context of human rights and it constitutes a starting point for the reflection on social phenomena connected with medicine. Taking into account the changes in medical ethics and culture which have taken place in the recent decades the question has been asked—is it worth talking about the right to be ill these days? Giving positive answers to this question, the spheres and issues that have been presented can be analyzed and assessed from the perspective of the right to be ill.

## Introduction

The right to health is nowadays understood as the unquestionable right of every human being. On the other hand, ‘the right to be ill’ may seem an absurd wording, since, as we think, the rights are supposed to advocate good. To be healthy is a common wish—disease is the evil which must be eliminated. Yet, all the questions connected with this issue are not preposterous and it is not only about freedom of decision about one’s own life. The idea of ‘the right to be ill’ was first presented and popularized many years ago by Tadeusz Kielanowski (1905–1992), an eminent Polish physician and moralist.

During the celebration of the 150th anniversary of French National Academy of Medicine in 1972 Kielanowski delivered a lecture ‘Le droit à la santé et le droit d’être malade’ (1972a), whose message was advocating of social and civil rights of the diseased and the disabled. He claimed the modern era had implemented the postulate of the right to health formulated as early as in the nineteenth century, whose constitutive part had become health insurance. He pointed out it was time to stand up for the rights of the diseased and the infirm to full participation in the life of the society and to deal with various manifestations of intolerance, discrimination and marginalization. Thus, as well as the right to health, the right to be ill should also be universalized. These issues were also raised in his articles: *The right to be ill* (1972b), *Medicine does not promise paradise* (1977), *Health of a Man* (1974), *Professional medical ethics* (1971).

Indeed, ‘the right to health’ was a great social achievement. It was not only a moral right, but also a constitutional one and enshrined in the international acts, starting with *The Universal Declaration of Human Rights* (art.25) and currently in numerous documents, e.g. in the *Charter of Fundamental Rights of the European Union* (art.35).

Fundamental changes in medicine, public sphere and health policy have taken place since Kielanowski’s publications. It is worth considering whether the right promoted by Kielanowski was merely an interesting but purely rhetorical device, or focused on the existing problems. But the question is also if today, in the face of many threats, the widening of the idea of human rights is justified.

It seems that this idea allows to view many issues from different perspectives and might be an inspiration even today. This article is an attempt to systematize ‘the right to be ill’ and it focuses on its presentation, tracing its origin, justification and some of its contemporary references.

## Kielanowski’s idea of ‘the right be ill’

Most theorists of rights would critically approach the term ‘the right to be ill’, because laws protect what is valuable to man, the fundamental values. It seems that the use of the phrase ‘the right to ...’ when referring to what is not good or positive is not justified. It could be argued that illness, in certain rare circumstances, can be a ‘good’. Of course, from the perspective of the diseased during the period of the sickness, it is always connected with suffering: physical, mental, spiritual. However, if you look from the perspective of the ‘whole’ of life, illness can sometimes be of value: it can help to reexamine one’s life and the past hierarchy of values, or get closer to God. Illness and suffering can be linked to personal development and an axiological look at one’s own weakness and pain. However, we enter here the sphere of the philosophical and theological sense of suffering—and this is not the aspect that Kielanowski had in mind in the formula of the law of sickness, and it is not the subject of this article.

What is, then, the meaning of the expression ‘the right to be ill’?

In one of his lapidary wordings (1972b, p. 3) this right means ‘the right of sick and weak people to live in society and not only on the fringes of the community’ and it states that ‘the sick man […] who does not work and does not produce anything is not by the very fact of his illness a second-rate citizen and does not deserve to be discriminated in any way’. The suffering related to the disease, disability or old age which people experience is sharpened by loneliness and isolation, or even intolerance in the first place, but also by different obstacles since the social sphere is shaped with the healthy, strong and fit in mind and that can lead to exclusion and degradation.

The formula ‘the right to be ill’ has thus a broad and substantial symbolical meaning, connected with the right to be weaker, to be different—the departure from the commonly accepted standards of health and fitness created by contemporary culture and also the right to self-determination. Indeed, it is not the “illness” itself that is the subject of this law, but all the laws and everything else that relates to being ill. It should not be understood literally, in the legal sense, but as an idea to raise awareness of important issues and to indicate the direction of solutions.

The semantic scope of this law encompasses two main dimensions: individual—referring to the so-called ‘negative’ nature of rights, i.e. freedom (using terminology from the domain of rights) and social—referring to the ‘positive’ nature:

1. In the individual aspect it meant mainly ‘the freedom of being ill’, the right to refuse to start or continue a therapy (excluding exceptional cases posing threats to the society). Thus, Kielanowski (1985, p. 8) emphasized that ‘apart from all the political and social liberties, freedom of opinion and freedom of speech [a person must have] also the right to be ill’. Kielanowski questioned this so-called ‘compulsory treatment’ particularly in his publications from the 1950s.

Emphasizing and promoting of ‘the right to health’ is socially beneficial, it is enough to present a few utilitarian arguments to justify it. Contrarily, the right to be ill is opposed to the rule of social advantage. This issue refers us to the problem of a more general nature—the relations between the individual good and the public good (Kielanowski 1971, p. 179).

2. In his publications from the 1970s Kielanowski concentrated particularly on the social dimension of the right to be ill, which is here perceived as the rights of the diseased, the disabled and the old. He points to the need of raising awareness and public concern to provide a public space for the diseased, the change in the way of thinking and in the human relationships (which might be understood as its horizontal application). He observes that nobody is allowed to be discriminated because of their disease, disability or dysfunction, moreover, he strongly recommends active tolerance which is ‘searching for and finding a place in the society for everyone, also for those who either physically or mentally differ from the norm and all the average values conventionally acknowledged to correspond to the notion of being healthy according to the utilitarian criteria (Kielanowski 1974, p. 174).

Secondly, this right requires a proper organization of social life, legal regulations and concern of the state (which would correspond to the vertical application)—so that ‘joy and bliss are not exclusively the privilege of the healthiest and the strongest, so that weaker people and the disabled too, will never and nowhere be discriminated against, that the gates of universities will not be closed to them, or the theaters, or state frontiers, or places of entertainment’ (Kielanowski 1972b, p. 4).

Why did Kielanowski point to the need of promoting the right to be ill? What potential threats did he see and what was it supposed to be the weapon against?

Kielanowski (1971, 1972a, b, 1974, 1977) presents himself as a critic of the excessively reductionist biomedical model of medicine and the subsequent implications and trends in the contemporary culture. Among numerous critical statements it is worth pointing out the ones which remain up-to-date and referring to:

- The problems of defining health adequately. Focusing on the perceiving disease as the deviation from the relevant norm, it determined the arbitrary perception of pathology and overlooking the psychosocial factors;
- The predominant model of technologically-oriented medicine, which has become mainly remedial medicine;
- Constantly reappearing utopian visions of the world without diseases;
- Designing and organizing community life (work, recreation, architecture, public transport, etc.) according to the needs of the healthy majority in mind;
- Increasing dependence of social life and human world of values on the principles of economics and utilitarian calculation;
- Dominating cultural patterns—of physical appearance, fitness, healthiness and strength as the only desired ones.

According to Kielanowski, the above-mentioned phenomena are related to, or result in, a kind of health worship—perceived mainly in its biological aspect, excessive expectations towards medicine and marginalizing people who are chronically ill, disabled or infirm; and they have a crucial relation to one-sided perception of the idea of human rights and the inadequate interpretation of the right to be healthy.

## Human rights and ‘the language of rights’

The aim of the rights is the protection of values which are significant for people—life, freedom, property, health, etc. The rights protect us against persecutions, unequal treatment, intolerance, invasion of privacy, but they also enable us to demand certain actions on the part of the society or the state. There is a basic division of rights into the rights guaranteed by the law (legal rights) and moral rights, of which common human rights are a part. ‘The right to be ill’ should be obviously considered in the context of moral rights being a foundation of social practice.

Nowadays, mainly in the Western world, it is characteristic not only to use ‘language of rights’ on the regular basis, but even overuse it—not only do we speak of the human rights in general, but also of children’s rights, patients’ rights, customers’ rights, as well as animals’ rights. And individual liberalism points out that rights should constitute a foundation of every moral or political theory. On the other hand, some critics call the idea of human rights ‘a modern ideology’ and they talk about the imperialism of human rights which have been imposed on the world by the Western culture (Osiatyński 2009; Glendon 2001). While it is true that the idea of human rights arouses controversy and is justified in different ways, e.g. by appealing to ideals such as equality, autonomy, human dignity, fundamental human interests, the capacity for rational agency, ‘minimally good life’ or democracy, it would still be difficult to imagine the modern world without human rights (Freeman 2004). They played a crucial role in its formation and they are one of the keystones of protection of individual interest and international politics.

In spite of the complexity of the subject of rights, it is worth focusing on certain basic distinctions. The rights are generally understood as legitimate claims submitted by individuals or groups against other people or the community. To have a right to something is to be able to state freely what others should and what they should not do (Hart 1973), thus, the rights are justified demands urging others to act or refrain from actions and that is why there is this distinction between positive and negative character of rights.

Certain rights assume some specified obligations—there is a significant but not always close relation between rights and obligations. So-called ‘positive rights’ assume someone’s obligation to ensure that a person in question will receive what is guaranteed by the right; therefore, it requires taking some action. The ‘negative right’ involves refraining from a certain action. Yet, these rights have two dimensions (both positive and negative) in most cases—thus, ‘liberties’, traditionally considered to be negative rights, also involve some obligations, e.g. they commit the state to passing the acts protecting these rights. The question who should bear the obligations remains contestable; in the traditional conception it is the responsibility of the state government (the vertical effect), but the rights also impose some obligations on certain groups and, consequently, on every individual (the horizontal effect of rights – referring to human interactions).

The right to health is mainly positive as it obliges the society to protect health of the citizens (it is chiefly the state’s responsibility which is to provide healthcare system). But also the negative aspect of this right might be signalized: a demand to refrain from actions harmful to health of an individual (both vertically and horizontally).

Are there certain rights imposing some obligations on the subject of these rights as well? Hence, does the right to health oblige us to care about our own health? And can others (the society) require and enforce it in any way? And, for example, the right to life—is it an obligation to preserve one’s own life? It would seem it is not, since one of the basic tasks of rights and liberties is protection of human freedom and as Dworkin (1978) put it—the rights are a trump card of individuals.

And this is generally how this issue is percieved by the law—but it can be viewed in a different way from the moral standpoint. The rights provide us with opportunities of fulfilling different needs and values such as life, freedom, health. If we perceive a human as a subject attempting to realize his potential in full and, at the same time, as a social being interacting with others and having responsibilities for and towards others, in moral meaning at least some of universal rights impose obligations on us. The right to health involves caring for one’s own health and the right to life means refraining from careless actions putting one’s life at risk. However, the problem is subtle here; this self-commitment is more of internal autonomy of a person as a moral subject. Yet, no one can compel us to such actions or put them into execution.

Philosophical controversy is also aroused by the answer to the question if the rights are unconditional and absolute. Some of them seem to be in fact unconditional (e.g. the right not to be tortured), but there might be clashes between the claims of individuals or groups and the common good. As a result, a quite commonly adopted solution is to consider most of the rights to be absolute *prima facie* (with reference to the theory of obligations by W.D. Ross)—which requires further procedures of balancing and elaborating on them (Ross 1930/2002; Beauchamp and Childress 1994). And it is not an entirely acceptable solution.

Referring the above-mentioned to the idea presented by Kielanowski—he spoke of risks stemming from the excessive concentration on some rights and their one-sided interpretation, which contributes to drawing precipitate conclusions about obligations and, in fact, leads to misusage. These risks made him speak of not only ‘the right to be ill’, but also the right to death (he was one of the first to raise this issue in writing) and the right of the patient to be not informed—it is reasonable to refer to them as a whole to show his line of reasoning.

The point is that in the social consciousness the understanding of the nature of human rights was the following: (1) imposed obligations (at least moral ones) on the subject of rights, which could be executed and failure to keep them would involve social sanctions, (2) treated the rights as unconditionally binding or (3) did not make a distinction between the negative and positive aspect of rights. As a consequence, the right to life would oblige people to preserve their lives in every situation, and, with respect to medicine, the obligation to keep a patient alive regardless of their condition. The right to truth would make the diseased person obliged to be fully informed about their condition and prognosis, and unconditional obligation for the physician, regardless of the situation, to inform the patients about all the details connected with the disease. The right to health—even the unconditional obligation to undergo a therapy and care about one’s health, which could even be subject to social sanctions. And consequently, it would result in a specific ‘health cult’.

In order to prevent abuse or unwanted consequences, which such an interpretation encourages, according to Kielanowski, the above-mentioned rights should be balanced or supplemented by their antitheses, indicating the limits that must not be crossed.

Thus, in order not to allow the overinterpretation of such an important right as the right to life to lead to e.g. futile medical care (overzealous treatment), which does not let the patient die ‘naturally’ causing only unnecessary suffering (Kielanowski 1975), it should be balanced or limited with the right to death.

In order not to allow the patient’s right to truth to function in practice as ‘professional’ but dry information, especially when it comes to poor prognosis, ignoring the incurably sick person’s vulnerability and emotions, it must be balanced with the right to be not informed (Kielanowski 1984), as the patient might not wish to know the details of the health deterioration and potential suffering (this right was later expressed in the Convention on Human Rights and Biomedicine, art.10). To prevent the right to health from marginalizing or discriminating the diseased, the disabled or those who give up the therapy, and not to make it contribute to excessive expectations from medicine and healthcare, it should be balanced with the ‘right to be ill’. Combination of these seemingly ‘opposing’ rights guarantees that neither of them will erode into a must to be healthy nor a passive giving in to a disease and social mechanisms of marginalization. This antithetic, dialectic method by Kielanowski indicates that the rights enabling us to fulfill our needs and potential must be a subject of limitations, and it refers mainly to positive rights. They are obviously unalienable (they cannot be taken away either by the state government or an individual), but this crucial quality cannot work against the subject of the rights.

## The question of substantiation of ‘the right to be ill’

For Kielanowski the practical meaning of the right to be ill was more important. Yet, some elements of the reflection over the background of this idea can be indicated. Kielanowski seems to agree with the view that every person is entitled to moral rights ‘by nature’, and as such they are universal and unalienable. Kielanowski liked the classic view of human rights, but he derived it rather from Kant’s view of an individual as an autonomous subject and purpose, and it was manifested in the interesting view of Hart, a contemporary influential philosopher of law—that if any universal moral rights exist at all, they are underlain by one fundamental ‘right to freedom equal for every man’ (Hart 1955). Hart’s viewpoint is mentioned here as Kielanowski has a similar attitude saying that every person should take advantage of their autonomy to such an extent which is compatible with another person’s freedom. This principal right to be an autonomous subject, the right to self-determination (Kielanowski 1978) is the source of every person’s moral commitment—the obligation to respect autonomy of others.

The present-day perception of human rights is the result of synthesis of two traditions: the Anglo-American ‘individualistic’ tradition underlining freedom of an individual (slightly diminishing the significance of limitations and responsibility) and the dignity-based tradition dominating in Europe (Glendon 2001) having its roots in the community. In Kielanowski’s approach one can observe more ‘freedom-based’ than ‘dignity-based’ character of rights. Although he seldom explicitly refers to the concept of personal dignity, focusing on the positive aspect of the right in question manifests his call for equal respect for every person as a self-determined subject (Kielanowski 1978).

Thus, the foundation of the right to be ill is twofold. On one hand, there is the autonomy giving the right to be healthy or ill. From the physician’s point of view health and life are basically the patient’s good, but for the patient it might mean something else—it is the patient who decides what is the most valuable thing: ‘the person’s good must remain what they consider their own good’ (Kielanowski 1985, p. 9). Other qualities—dignity, freedom or being faithful to one’s convictions might be more important than health or even life.

On the other hand, particularly in the social dimension of this right, it is underlain by dignity and community perspective. Here, for Kielanowski the substantiation seems to be a universal and rudimentary ethical rule, ‘the rule of reciprocity’. That is why he writes ‘it is worth being constantly repeated that the infirm, the chronically ill or the disabled are not some unspecified »them«, but in fact they are one of us’ (Kielanowski 1977, p. 48). Therefore, it results from the very fact of being human that ensuring ‘the right’ to be ill should stem from the sense of justice and not from pity (Kielanowski 1972b, p. 4). Human health is not purely biological concept and the value of life can be measured in economic or utilitarian categories. Hence, an ill or disabled person should take advantage of all the rights unreservedly ‘not because this person could be potentially useful or productive, but simply because of being human. The right to work, entertainment, taking actions, participating, having fun results from this, so it cannot be the act of mercy or charity (Kielanowski 1974, p. 174).

Kielanowski substantiates ‘the right to be ill’ not only in the moral sphere, which is the most significant here, but he also offers natural and medical arguments. He starts from the point that a disease is a natural phenomenon, all organisms are taken ill: it is a symptom of life whose significant feature is the state of dynamic balance of the living system (homeostasis) and the disease is its disruption. Referring to the dynamic-adapting notion of health by Rene Dubos, he views a disease as an adaptation effort of an organism or an adaptation conflict; medicine is to help in this conflict, by aiding to regain lost balance using proper means. A disease is at the same time the beginning of the process of recovery, so building a new homeostasis (Kielanowski 1974, p. 173; Bilikiewicz and Kielanowski 1964, p. 21). Thus, the vision of the world without diseases—despite long-time human craving—is obviously a utopia and chimera.

Secondly, without deviations from ‘the norm’, progress is not possible. Diseased communities are the communities capable of further evolution—as opposed to social insects, intolerant of sick individuals and unchanging and stabilized for millions of years: ‘so this is also one of the reasons—as Kielanowski writes (1972b, p. 4)—why we do not wish to abandon individuals who are peculiar both physically or mentally, and the diseased’. Particularly in the human world, diversity, also biological one, is the quality which underlies the development of culture. Prominent artists are often quite far from the standards of humankind, suffering from various dysfunctions or diseases. And this very distinctness or disease and sufferings involved play also an important role in their creative work (it is enough to mention Chopin, Schiller, Beethoven, van Gogh, Keats, Proust).

## What must be said about ‘the right to be ill’ today

Was Kielanowski’s concern justified and should we also talk about ‘the right to be ill’ today? The answer would require a thorough consideration of many aspects of this voluminous idea. Some of the issues which can be assessed and analyzed from this right’s perspective can only be hinted at here.

In its negative dimension, that of ‘freedom to be ill’, it was Kielanowski’s particular response to the implications of the authoritarian healthcare system, manifestations of unjustified paternalism and the practice of the so-called ‘compulsory treatment’—the use of different forms of pressure on the patient so that they undergo the indicated form of therapy. In this sphere, the recent decades have brought about significant changes—thanks to both the patients’ rights movement and bioethical discourse, for which autonomy and the principle of respect for autonomy have become one of the most fundamental. The obligation to respect the patient’s autonomy has sensitized medicine to the problem of the patient’s consent to medical procedures and now the necessity of obtaining an informed consent is basically a standard. The developed notion of informed consent to healthcare interventions formulates the conditions stating that we can talk about giving it if the patient is competent in his actions (capable of taking decisions), in possession of all relevant facts, comprehends them, acts on the voluntary basis and agrees to an intervention (Beauchamp and Childress 1994). Although each of these conditions is disputed as for their content and range, this principle is one of the most significant instruments of the protection of the patient’s autonomy. It must not be idealized or treated in a purely formalistic manner, and the principal aspect of informed consent should be a proper communication between the physician and the patient and the patient’s comprehension of the situation. The idea of the right to be ill indicates that the final decision on whether to undergo therapy and in what form belongs to the patient.

However, freedom to be ill has some limitations concerned with practicing certain forms of coercion in order to preserve health. It pertains mainly to compulsory vaccinations, diagnostic examinations, certain contagious diseases and mental illnesses. Since constraint violates autonomy and privacy, it is necessary to give its justification. In case of dealing with contagious epidemic diseases which pose a threat to the society it is fairly easy to justify compulsory treatment, hospitalization or diagnostics. However, there are often controversies around compulsory diagnostic examinations or preventive vaccinations, which is manifested by protests against them. Is it fair, following the right to be ill to give an individual a freedom of choice? This problem is usually solved in favor of the common good (whose depositary is every member of the society) on the basis of different ethical principles e.g. justifiable paternalism or Thomas Aquinas’ Principle of totality. Nevertheless, it seems that the dilemma of medial totality does still exist as the conflict between the common good and the subjective good of an individual cannot be conclusively settled—however, a reasonable consensus is always possible. Kielanowski indicated here that the right to health and the right to be ill should be publicized and executed side by side—the collective have a right to be healthy; actions for the sake of public health are justifiable and necessary and the freedom to be ill has its boundaries which are delineated by liberties and rights of another person—the harm which this individual might experience now or in future as a result of their violation. However, conflicts are practically inevitable, which also results from the language of rights.

The right to be ill gains in a particular significance when it comes to mental illnesses due to the controversial nature of psychiatric treatment, questioning of ‘the real’ character of mental illnesses by some authors, hospitalization of the patients who do not feel ill or deny being ill. This problem is even more morally significant and delicate as the compulsory treatment in psychiatry often turns into the use of force. Here, again, the right to be ill will remind us about the necessity to respect the patient’s subjectivity and autonomy and applying, to the due extent, the principle of the patient’s consent to therapy and hospitalization. It is true that since Declaration of Hawaii, and even more so since Declaration of Madrid (WPA 1996) the ethical part of mental illnesses treatment has been given its rightful place; emphasizing the standards of obtaining consent to starting the treatment and limiting the use of force to emergency cases such as life-threatening situations for the patient or other people involved. Yet, the abuses occurring in psychiatric practice make us keep a vigilant eye on the question of respecting the patient’s rights and liberties.

What is the extent of the right to be ill as far as human life is concerned? It seems that it reaches its end—since it would be (combined with the right to death) a good substantiation of provision making for healthcare decisions in the event that, in the future, he/she becomes unable to make those decisions, so-called advance directives. Taking them into account one respects treatment refusal at each stage, the option of not giving consent to life-saving or life-sustaining procedures. Advance directives used in practice, facilitating reducing the suffering connected with dying prolongation are now popular in many countries, in different forms of written guidelines (a living will, a description of acknowledged values) or giving someone a durable power of attorney for healthcare. But, the fact that such procedures are not commonly used confirms the fact that there are some problems involved—how (and if in a different manner) would the person leaving the directives decide being in a given extreme life-threatening situation and unable to make decisions—would they be willing to change their directives if they had the power to decide?

The idea of the right to be ill opens a new interesting perspective for the critical analysis of contemporary phenomena—medicalization, healthism, human enhancement—the increasing trends noticed by Kielanowski, which were to be counterbalanced by this right. Medicalization, as a process of expansion of medicine, which controls vast spheres of life (from controlling basic functions of human organism to social life) leads to naming and perceiving many of them in medical terms and ‘treatment’ is seen as the remedial action. The picture of medicalization presented by Illich in the 1970s was exaggerated—but it proposed an important discourse about the social role of medicine: just as Foucault’s critical analyses along with his power/knowledge, biopower and biopolitics indicating, among other things, that medical discourse was not only to define a disease but also sanction normality or abnormality of an individual (Foucault 1971), and medicine being increasingly entangled in the state mechanisms has become the institution controlling social life by defining, normalizing, introducing discipline, unifying and regulating.

Contestatory nature of the right to be ill manifests itself particularly towards negative symptoms of healthism. In the view of Crawford (1980), the author of the notion of healthism, in the western culture health has become to be seen as a pervasive value—determining identification of all the values of life with health; at the same time a growing number of behaviors and approaches are evaluated by their health implications and labeled as ‘healthy’ or ‘unhealthy’—which categories are defined by medicine and show the proper life style. The concept of individual responsibility, pivotal for healthism indicates that life is mainly dependent on personal concern and choices—becoming a subject to moral assessment. Health is within one’s range, the one who is not healthy is to blame. This individual responsibility for health is deepened by the idea of risk becoming fundamental in the description of the medical condition of the society, and the notion of risk is made a key element of the health education language. Its individualized factors—smoking, being overweight, sedentary life style, etc. have started to be identified with a disease itself (Blaxter 2004).

Healthism is shaped by many factors including a phenomenon of aestheticization of everyday life specific to postmodernism, here mainly concerned with the human body which has become a special focus of attention. The body, which has started to express identity of an individual, is currently given a thorough insight and ‘the project of oneself has become a design of one’s body’ (Chrysanthou 2002). According to Giddens (1991) the more post-traditional the social conditions are, the more forced we are to design it. The involvement of medicine in this process is more and more significant—as health is manifested by a beautiful body and becomes its parameter. Thus, health obtains also aesthetic value—sometimes it is even straightforwardly identified with aesthetic qualities of the body (Fox 1993). The qualities highlighted in particular by the media, advertising industry and fashion are gaining importance: the body shape, attractive looks, being slim, physical fitness and sexual attractiveness. Dominating cultural standards of beauty fuel the pursuit of a beautiful body—fit and forever youthful. Properly shaped and conditioned it seems to be the way to success and happiness, as a carrier of pleasure in consumer culture (Featherstone 1991). The need to body care imposes certain life styles, thanks to which a contemporary man wants to be free from suffering, ugliness, disability, old age. This purpose is served by suitable dietary regimens, gymnastic routines, pharmaceuticals and cosmetics, medical procedures, especially plastic surgery.

On the other hand, everything which deviates from the standards of fitness and good appearance is stigmatized as a disease, the synonym of ugliness in the aestheticizing perspective of healthism. Health and disease get and additional symbolic meaning reinforcing their moral assessment. Being unhealthy sometimes is even perceived as individual moral license (Kirk and Coloquhoun 1989). Already in Sontag (1978) reflected on the figurative associations of disease with values. Some diseases are identified with everything which is culturally not accepted and defined as being ugly, impure and epitomizing evil—becoming a taboo and marking the realm of fears.

From the perspective of the right to be ill the concern of ‘the body project’ is a matter of personal choice as it authorizes the principle of respect for autonomy, the right to be different and diverse. The contemporary aestheticizing tendency is a source of stereotypes and resulting labeling and stigmatization of those who do not conform to cultural standards. All the more so, since the body aesthetics is more concerned with looking healthy than being healthy; beautifying medical procedures—liposuction, wrinkle reduction treatments, breast implants, etc. serve the purpose of masking a biological body degradation. Health—disease, beauty—ugliness, moral good—evil belong to different categories of values. Mixing medical aesthetic and moral classifications results in blurring the world of values, and, as a consequence, in its destruction. Figurative meanings of health and disease are justified in the artistic expression—in reality they do not have anything in common with moral or aesthetic assessment.

The right to be ill does not eliminate individual responsibility for one’s health, but objects to putting the moral blame on the patient; and with reference to the idea of risk, it takes into account its probabilistic nature. There is a clear line between a positive promotion of health and education and the development of preventive medicine, and overusing health rhetoric for other purposes including business goals of pharmaceutical and cosmetic industries. At the dawn of medicine a disease was often perceived as a punishment, especially for sins. These days the idea of punishment is being updated—becoming a sort of bad karma, retaliation for the wrong life style, irresponsibility for one’s own life, ignoring dietary regimens, medical check-ups, genetic tests, incorrect parents’ decisions. The right to be ill objects to this fatalistic and deterministic way of thinking. Placing too much responsibility on an individual also results in freeing politicians or healthcare specialists from their answerability, potentially blocking the necessary reforms (Blaxter 2004). Excessive concentration on the idea of risk may lead to ridiculousness and our life may become a nightmare if we are under its constant pressure—counting calories or avoiding HIV-positive people.

In the social dimension, the idea of the right to be ill opposed marginalization and social exclusion of the disabled, as well as the chronically ill and the old—it pointed to the necessity of protection of their rights and expressed concern about reintroducing them to the society. Certainly, we need to see the difference between disability and disease (considering their influence, especially in the case of long-term diseases, in developing impairments). However, in the early 1970s when Kielanowski wrote about this idea, in the popular discourse disability was seen in the biomedical categories—focusing on defects, flaws, dysfunctions of the organism—and it was symbolically approached as a ‘personal tragedy’ of an individual. It was perceived as the aftermath of a congenital defect, a disease or injury and from the medical point of view the patient was restored to normal life by means of treatment, medical rehabilitation or partial compensation for ‘deficiencies’(adjusting to the norms of community living). In extreme views disability was perceived as disease. The model concentrated on deficiencies and ‘rehabilitating’ along with the overinterpretation of the right to health assuming an obligation to be healthy, generated different forms of stigmatization and marginalization (particularly in the context of healthism), but also low self-esteem and inaction of the disabled, who were perceived as patients and subjects of medical treatment, and at the same time their image of being weak, helpless, dependent, regressive, strangely looking, etc. was reinforced (Zola 1993).

Kielanowski’s approach referring to the rights was one of important voices calling attention to the oppressive character of social barriers, stressing the significance of the cultural background of disability as a social construct and, beginning with medical understanding of disability, it criticized its medicalization and was an attempt to go beyond the medical context. It pointed to the necessity of social changes and changing the perception of disability, thereby contributing to shaping of a social model.

The concern about improvement of disabled people’s situation arose from many sources and clearly it has been intensified since the 1970s. With the significant participation of the disabled, strictly medical approach was criticized as one-sided and incomplete. One of the turning points, the manifesto Union of the Physically Impaired Against Segregation (UPIAS 1976) included a substantial distinction between : ‘impairment ‘as an impediment of organ functioning and ‘disability’ as an activity limitation and exclusion caused by the organization of society. It gave rise to new definitions and concepts, evolving into social models, indicating that injuries or deficiencies do not create disability in its social understanding (Morris 1993). It was thoroughly reflected in WHO documents: International Classification of Impairments, Disabilities, and Handicaps (WHO 1980) and International Classification of Functioning, Disability and Health (WHO 2001). The last, binding document is a combination of two opposing models—a medical (individual) one and a social one - as an biopsychosocial, interactive approach. It is shown that disability ‘is an umbrella term for impairments, activity limitations and participation restrictions. It denotes the negative aspects of the interaction between an individual (with a health condition) and that individual’s contextual factors (environmental and personal factors)’ (WHO 2001).

Both the definition, understanding of disease and health as well as medical or social perspective have influence on perceiving the relation between disability and disease. The followers of the biomedical approach and narrow definition of health as the absence of disease—which in turn is being defined as a ‘deviation from the functional organization of typical members of a species’, as in Boorse’s (1987) paradigmatic definition—will be prone to consider disability as a state of sickness, a state of being unhealthy. Interesting, in the field of this model, is a Amundson’s suggestion, who treats disease as an atypical process that tend to result in disability, pain, or death (Wasserman et al. 2016). Disability, which is not the result of this process is not the symptom of a disease—the disabled may be a healthy person. The supporters of more ‘holistic’ concepts have a different view on these matters as they adopt a broader definition of health as more, or other, than the absence of disease, as e.g. in Nordenfeldt’s (1995) perception, according to which health is the ability of an individual to fulfill all the ‘vital goals’, ‘those goals which are necessary and jointly sufficient for a minimal degree of happiness’. In this view of health as the instrumental value, it is vital to consider whether the impairment restricts or forbids attempts and accomplishment of individual goals; if not—then the disabled may be perfectly healthy.

However, regardless of the above views, bearing in mind the wide and symbolic meaning of the right to be ill, it placed the question of impairment in the social sphere and raised the question of rights and equality of the disabled. Simultaneously, with shaping the opinions of disability in terms of socio-political matters and developing to Disability Studies there was an increasing attention devoted to disability in the context of human rights. Theoretically, people with disabilities, just as all the others, are undoubtedly subjects entitled to rights and liberties. However, in practice it turned out that it is necessary to protect them against discrimination in using their rights and it contributed to creation of a number of documents and national and international legal instruments. Ratified in 2006 UN Convention of the Rights of Persons with Disabilities was an expression of the need to codify their rights placed in documents of different weight and making them binding. Aiming ‘to promote, protect and ensure the full and equal enjoyment of all human rights and fundamental freedoms by all persons with disabilities, and to promote respect for their inherent dignity’ (United Nations 2006: article 1) not only did it establish important definitions describing disability in social categories and present a comprehensive catalogue of rights, but also defined the main principles on which it was based: respecting dignity, autonomy, freedom of choice, indiscrimination, social integration, respecting differences, equal opportunities, accessibility. It is interesting that these rules could be easily derived from the idea of the right to be ill.

In the light of the UN Convention and the EU documents the normative process of the rights of the disabled is really advanced, which indicates that the right to be ill has set a right direction. Today—from the perspective of this right, in its social context—the crucial challenge is to make the accepted standards effective as it is not enough to give the disabled a bundle of rights or preferences equalizing their opportunities. It is pivotal for these people to want and be fully able to make use of these rights, which requires not only formal, but also actual acceptance. Are we really creating a friendly setting for everyone and integrating the disabled in the regular daily life?

This question refers also to the problem of old age, which is neither a disease nor disability but does correspond to them. The phenomenon of ageism, a discrimination of elderly people, frequently described and analyzed shows real social problems of senior citizens’ existence—and leads to a conclusion about the need to work out some instruments protecting subjectivity and the rights of the elderly. Here again the right to be ill turns out to be farseeing, which is confirmed by formation and work of The Open-ended Working Group on Ageing and the Global Alliance for the Rights of Older People, and in particular, the initiation of work on the convention on the rights of older people.

## The conclusions and prospects

The right formulated by Kielanowski was grasping problems and clashes appearing in the relation an individual—medicine—the society. The right to be ill encompasses two dimensions: individual, as a ‘freedom to be sick’, and social (the right of people who are ill, disabled or old)—horizontally and vertically—indicating a necessity of proper organization of social life, of creation of legal instruments and of concern of the state. All in all, this right features vast autonomy in matters referring to one’s own health and therapy, self-determination and respecting the rights of all persons who have problems with access to social life because of being ill or disabled, they find it more difficult to pursue their goals; and they become marginalized or excluded.

Since Kielanowski’s publication, there have been a lot of fundamental changes in line with this right (mentioned in the previous section)—and the reason for this is that it corresponds to the notion of human rights upholding the value of a person, dignity, subjective autonomy, equality and social justice. The idea of the right to be ill can be understood as the quintessence of many laws.

Is it worth, then, to refer to this idea and what would its role be today?

Perhaps above all, it broadens the prospect of insights into the moral and social issues connected with disease and health. It may become an additional validation for already-mediated solutions (as a result of many discussions) and standards in medical ethics (e.g. informed consent, advance directives) and for patient rights. On the other hand, it constitutes an important reinforcement of the changes in perceiving people with incurable illnesses, with disabilities or old—consolidating the principles of respect for their subjectivity, understanding and acceptance of their individualized needs, perceiving them as full members of the society—and in creation of adequate instruments for reforming and specific solutions in social practice. Nevertheless, it can serve as a criterion and a tool for the assessment of the social awareness evolution and implementation of prepared documents.

The right to be ill is not in opposition to the right to be healthy—it is rather its consequence, the opposite pole. It presents a natural dynamics of the process of life ranging between health and disease, fitness and infirmity, the youth and the old age, life and death. Modern history shows that if a disease, disability, old age, death are seen as a failure of medicine, not meeting the expectations, in spite of its scientific and technological achievements, the very essence of medicine is distorted and it becomes a mere health technology. When medicine focuses on the disease in its biological aspect and is mainly remedial—it leads to reductionism, dehumanization and depersonalizing of the patient. The concept of the right to be ill seems to restore balance in different trends of medicine and its social context. The critical feature of this right calls for the need of continually repeated analysis of the directions of medicine development and its fundamental notions, goals and priorities—especially in the light of growing social expectations towards medicine (along with the financial shortage); a valuable example of which was an international project ‘The Goals of Medicine. Setting New Priorites’(Callahan 1996).

On the other hand, when thinking about the problem in terms of ‘health’ and intensification of its promotion, which is a valuable achievement of recent decades, it turns into dictatorship obliging people to be healthy and, by treating health as a highest, autotelic value, it leads to idealization of health—and to healthism—and makes health a commercial commodity. Intellectual trends give it a moral and aesthetic undertone, creating cultural stereotypes of health, physical appearance and fitness, increasing public demand for body correction as well as utopian projects of human enhancement, for which genetic engineering advancements open up completely new horizons. Socially favored patterns encourage labeling and stigmatization of those who are different. The concept of the right to be ill can play an important role in a discussion with healthism, as it expresses, inter alia, the idea that health is not an all-encompassing and moral imperative. It shows the value of health as instrumental for the realization of human goals.

Arguing for the meaning and utility of the concept of the right to be ill, it is worth noting, in addition to the above, the psychological, ‘therapeutic’ meaning of this right—shaping the perception of self and others, as a disease is not only a biological state of one’s organism. The person who is chronically ill, disabled or elderly must reconsider his or her goals, which is a source of various fears, the state of helplessness, insecurity. The right to be ill makes a person aware that ‘I have the right to be ill’, feel unwell, not to be able to cope; it allows to accept a ‘disease’—which does not mean being passive or giving in—on the contrary, in struggling with a disease it is pivotal to be able to cope and use different ‘resources’. Antonovsky (1987) in his model of salutogenesis pointed to the significant role of a continuous variable - the sense of coherence (SOC) as a relatively constant attitude determining the perception, feeling and comprehension of self in the world and having three components: comprehensibility, manageability and meaningfulness. And in these components the awareness of the right to be ill might play an important role. Perception of the world from the perspective of ‘this right’ indicates that a disease, weakness, being different or infirm is not an unusual or absurd state; they are components of ‘the whole’, the system of life. It can protect us against negative emotions or the sense of helplessness. It carries a message that life is meaningful after all, it is worth the effort and motivates to action; this motivation is connected with positive self-experience. Subjectively, it restores a sense of dignity and self-respect in a person who despite unfavorable circumstances can make decisions about his or her life. This psychological aspect has a broader meaning: every person has a right to periods of feeling unwell or unfit, which can make fulfilling obligations impossible or difficult. It is not a trivial matter as it might be confirmed by the phenomenon of ‘sick presenteeism’.

In the context of social references the right to be ill has a particularly anti-discriminatory character and it states: ‘you/they have the right to be ill’. Thus, it orders to accept another person as a patient, focusing on shaping individual and social sensitivity and countering exclusions. The rights assume some obligations—so it results in the duty of active acceptance, manifested in social life, which is in turn associated with the whole context of positive nature of the right to be ill.

Created declarations and legislations are necessary instruments used to enforce the rights and claim them. However, without a change of attitudes in the sphere of acknowledged values, they will remain mechanisms, efficient as they might be, but without deeper essence—and introducing these changes is one of the essential aims of the idea of the right to be ill.

## References

[CR1] Antonovsky Aaron (1987). Unraveling the mystery of health—how people manage stress and stay well.

[CR2] Beauchamp Tom L, Childress James F (1994). Principles of biomedical ethics.

[CR3] Bilikiewicz Tadeusz, Kielanowski Tadeusz (1964). Czynniki psychiczne w zdrowiu i w chorobie (Psychological Factors of Illness and Health). Zdrowie Publiczne.

[CR4] Blaxter Mildred (2004). Health.

[CR5] Boorse Christopher, VanDeVeer Donald, Regan Tom (1987). Concepts of health. Health Care ethics: an introduction.

[CR6] Callahan Daniel (1996). The Goals of medicine: Setting new priorities. Hastings Center Report.

[CR7] Chrysanthou Marc (2002). Transparency and selfhood: Utopia and the informed body. Social Science and Medicine.

[CR8] Crawford Robert (1980). Healthism and the medicalization of everyday life. International Journal of Health Services.

[CR9] Dworkin Ronald (1978). Taking rights seriously.

[CR10] Featherstone Mike, Mike Featherstone Mike Hepworth, Turner Bryan S (1991). The body in consumer culture. The body: social process and cultural theory.

[CR11] Foucault Michel (1971). L’ordre du discours.

[CR12] Fox Nick (1993). Postmodernism, sociology and health.

[CR13] Freeman Michael (2004). Human rights: An interdisciplinary approach.

[CR14] Giddens Anthony (1991). Modernity and self-identity: Self and society in the late modern age.

[CR15] Glendon Mary Ann (2001). A world made new: Eleanor Roosevelt and the Universal Declaration of Human Rights.

[CR16] Hart Herbert LA (1955). Are there any natural rights?. The Philosophical Review.

[CR17] Hart Herbert LA, Simpson Alfred WB (1973). Bentham on legal rights. Oxford essays in Jurisprudence.

[CR18] Kielanowski Tadeusz, Sarapata Adam (1971). Zawodowa etyka lekarska (Professional medical ethics). Etyka zawodowa.

[CR19] Kielanowski Tadeusz (1972). Le droit à la santé et le droit d’être malade. Bulletin de l’Académie Nationale de Médecine.

[CR20] Kielanowski Tadeusz (1972). The right to be ill. Poland.

[CR21] Kielanowski Tadeusz (1974). Zdrowie człowieka (Health of a Man). Człowiek i nauka.

[CR22] Kielanowski Tadeusz (1975). Medyczne i moralne problemy umierania i śmierci (Medical and moral problems of dying and death). Etyka.

[CR23] Kielanowski Tadeusz (1977). Medicine does not promise paradise. Poland.

[CR24] Kielanowski Tadeusz (1978). Prawo człowieka do stanowienia o sobie (The right of a man to decide about himself). Człowiek w obliczu śmierci.

[CR25] Kielanowski Tadeusz (1984). Mówić prawdę! Mówić prawdę...? (To tell the truth! Or shall we?). Służba Zdrowia.

[CR26] Kielanowski Tadeusz, Kielanowski Tadeusz (1985). Wprowadzenie do nauki o etyce i deontologii lekarskiej (Introduction to medical ethics and deontology). Etyka i deontologia lekarska.

[CR27] Kirk David, Colquhoun Derek (1989). Healthism and physical education. British Journal of Sociology of Education.

[CR28] Morris Jenny (1993). Independent lives? Community Care and Disabled People.

[CR29] Nordenfelt Lennart (1995). On the nature of health: An action-theoretic perspective.

[CR30] Osiatyński Wiktor (2009). Human rights and their limits.

[CR31] Ross, William D. 1930/2002. *The right and the good*. Oxford: Clarendon Press.

[CR32] Sontag Susan (1978). Illness as metaphor.

[CR33] United Nations (2006). Convention on the rights of persons with disabilities.

[CR34] UPIAS (1976). Fundamental principles of disability.

[CR35] Wasserman, David, Adrienne Asch, Jeffrey Blustein, and Daniel Putnam. 2016. Disability: Health, well-being, and personal relationships. In *The Stanford Encyclopedia of Philosophy*, ed. Edward N. Zalta. https://plato.stanford.edu/archives/win2016/entries/disability-health. Accessed 27 May 2017.

[CR36] WHO (1980). International Classification of Impairments, Disabilities, and Handicaps.

[CR37] WHO (2001). International Classification of Functioning, Disability and Health.

[CR38] World Psychiatric Association. 1996. *Madrid declaration on ethical standards for psychiatric practice*. http://www.wpanet.org/detail.php?section_id=5&content_id=48. Accessed 1 June 2016.

[CR39] Zola Irving K (1993). Self, identity and the naming question: reflections on the language of disability. Social Science and Medicine.

